# Retinol-binding protein 4 in combination with lipids to predict the regression phenomenon of autism spectrum disorders

**DOI:** 10.1186/s12944-021-01522-9

**Published:** 2021-08-26

**Authors:** Jianling Chen, Jing Chen, Yun Xu, Peipei Cheng, Shunying Yu, Yingmei Fu, Yasong Du

**Affiliations:** 1 Department of Child & Adolescent Psychiatry, Shanghai Mental Health Center, Shanghai Jiao Tong University School of Medicine, 600 Wan Ping Nan Road, Shanghai, China; 2 Department of Shanghai Key Laboratory of Psychotic Disorders, Shanghai Mental Health Center, Shanghai Jiao Tong University School of Medicine, Shanghai, China

**Keywords:** Retinol binding protein 4, autism spectrum disorder, Lipid, Cholesterol, Triglyceride, Autistic regression

## Abstract

**Background:**

About 20–40 % of autistic people experience a phenomenon of regression. Retinol binding protein 4 (RBP4) plays an important role as an inflammatory neurotrophic adipokine and is a promising mediator of the fat-brain axis. Abnormal fatty acid metabolism and lipid mediators have been reported to be related to the etiological mechanism in autism, and amelioration of impaired lipid metabolism can be recognized as a treatment strategy for autism. The purpose of this study is to explore the relationship between RBP4, lipids, and the autistic regression phenomenon, and to discuss their potentials as biomarkers for the autistic regression phenomenon.

**Methods:**

A total of 60 autistic individuals (18 with regression phenomenon, 42 without regression phenomenon) (ASD group) and 36 healthy controls were enrolled in this case-control study. The levels of RBP4, total cholesterol (TC), high-density lipoprotein (HDLC), low–density lipoprotein (LDLC), and triglyceride (TG) were measured. Childhood Autism Rating Scale (CARS) is used to assess the severity of autism. Ethical measures were performed in compliance with the current Declaration of Helsinki and written informed consent was obtained from the parents before enrollment of the children and adolescents.

**Results:**

Compared with control subjects, autistic individuals had lower levels of TC (*P* = 0.007), RBP4 (*P* = 0.001), and HDLC (*P* = 0.027). The levels of RBP4 in ASD group were positively correlated with TG (*r* = 0.355, *P* = 0.005), HDLC (*r* = 0.257, *P* = 0.047), TG/TC (*r* = 0.376, *P* = 0.003) and TG/LDLC (*r* = 0.363, *P* = 0.004), and were negatively correlated with CARS (r=-0.296, *P* = 0.003). Further logistic regression demonstrated that decreased RBP4 concentration was associated with the presentation of the autistic regression phenomenon even after the adjustment of the potential confounding factors.

**Conclusions:**

Serum RBP4 is associated with the autistic regression phenomenon and the severity of ASD. Further studies are needed to expound whether decreased RBP4 participates in the development of the autistic regression phenomenon.

## Introduction

Autism spectrum disorder (ASD) is mainly manifested in the deficits of social communication and social interaction, as well as repetitive behavior, limited or restricted interest or activity [1]. Epidemiological data indicate that ASD is a life-long disorder with an increasing prevalence and has become one of the most rapidly growing serious disorders in the world [2–4]. About 20–40 % of autistic children experience the regression phenomenon in language, social interaction, or motor ability after normal or relatively normal developmental stages [5–7]. Although the autistic regression phenomenon can occur at any age, the average age was 21.35 months (1.78 years), which is a high-speed period of neurodevelopment [8]. Children with autistic regression phenomenon have more cognitive deficits and more severe symptoms of ASD than children without autistic regression phenomenon [9]. In addition, the clinical identification of this regression phenomenon lacks specific biological or laboratory criteria and often relies on the history provided by the family and the observations of the clinician, which are highly subjective [10]. Therefore, searching for predictive biomarkers of regression in autistic individuals will be of great help for the early intervention.

The exact etiology and mechanism of the autistic regression phenomenon remain unclear. Current research assumes that it may be related to the interaction between biological and environmental factors [9, 11, 12]. For example, mitochondrial dysfunction, metabolic abnormalities, Rett syndrome, epileptic encephalopathy, over-pruned neural network, psychosocial stressors, prenatal and obstetric complications, viral infection, the socio-economic status may all be related to the regression phenomenon [12]. Retinol binding protein 4 (RBP4), a newly discovered adipose factor, is the main transporter of retinol and is related to the dysregulation of energy metabolism, insulin resistance, diabetes mellitus, and obesity [13–15]. Most recent studies have shown that RBP4 is permeable to the blood-brain barrier (BBB) and increases with the dysfunction of BBB [16]. In addition, RBP4 plays an important role as an inflammatory neurotrophic adipokine and is a promising mediator of the fat-brain axis [16].

Former studies have identified that plasma RBP4 levels both in the first trimester and second trimester are dose-dependently associated with increased risk of gestational diabetes mellitus (GDM) [17]. In addition, there is growing evidence for the role of GDM in the pathogenesis of neurodevelopmental disorders among which is ASD [17, 18]. However, whether RBP4 is associated with the autistic regression phenomenon is still unclear.

Additionally, abnormal fatty acid metabolism and lipid mediators have been reported to be related to the etiological mechanism in autism, and amelioration of impaired lipid metabolism can be recognized as a treatment strategy of autism [19–21]. Furthermore, researchers have experimentally shown that lipogenic enzymes, such as fatty acid synthase (FASN) and acetyl-CoA carboxylase (ACC), are highly expressed in the rodent brain during the early neonatal period and decline thereafter [21, 22]. This phenomenon of decreased expression of lipogenic enzymes may be reminiscent of the autistic regression phenomenon. Nevertheless, whether lipids are associated with the autistic regression phenomenon remains ill-defined. Therefore, the purpose of this study is to explore the relationship between RBP4, lipids, and the autistic regression phenomenon, and to discuss their potentials as biomarkers for the autistic regression phenomenon.

## Methods

### Participants

Autistic participants were 60 children and adolescents aged 3 to 14 years old, 39 males and 21females, the average age was 7.3 ± 3.2 years. Healthy control participants were 36 children and adolescents aged 3 to 14 years old, 12 females and 24males, the average age was 7.3 ± 3.1 years. Autistic participants were recruited from the outpatient department of child and adolescent psychiatry in Shanghai Mental Health Center between September 2018 and August 2019. All autistic participants were by the diagnostic criteria of autism spectrum disorders proposed by the Diagnostic and Statistical Manual of Mental Disorders, Fifth Edition (DSM-5). Healthy controls were recruited from the children who came to the same hospital during the same period for regular consultation but had not been diagnosed with any mental disorders. Individuals with Fragile X syndrome or other pervasive developmental disorders, obsessive-compulsive disorder, affective disorder or other neuropsychiatric disorders, neurodegenerative disorders, brain trauma or cerebrovascular disorders, metabolic disorders, or immunological disorders were excluded.

This study was performed in strict accordance with the Declaration of Helsinki and other relevant national and international regulations. All procedures for this study were approved by the institutional review boards of Shanghai Mental Health Center. Written informed consent was obtained from the parents before enrollment of the children and adolescents.

### Diagnostic and symptom assessment

Child Autism Rating Scale (CARS) was applied to evaluate autistic symptoms [23]. Total scores of CARS range from 15 to 60; scores below 30 indicate that the individual was non-autistic, scores between 30 and 36 indicate that the individual was mild to moderate autistic, and scores between 37 and 60 indicate severe autism.

Autistic participants were further assessed based on the following questionnaire [24] and were subdivided into Autism spectrum disorders with regression phenomenon group (ASD-R) and without regression phenomenon group (ASD-NR). Specifically, parents of all 60 autistic participants completed an intake questionnaire, which contained the following questions about regression:(a) Did your child experience loss of language skills for at least 3 months? (b)Did your child experience loss of the ability to spontaneously use at least five words? (c) Did your child experience loss of the will to communicate? (d) Did your child experience a loss of grammatical skills? (e). Did your child experience a loss of pronunciation skills? (f) Did your child experience loss of regular skills for at least 3 months? (g) Did your child experience loss of the ability to grasp or hold something? (h) Did your child experience loss of the ability to do a puzzle, play chess, or imitate? (i) Did your child experience loss of social relationships, interests, or participation? If the answer “Yes” is to any item of the above questionnaire, the corresponding participant was subdivided into the ASD-R group. Otherwise, the corresponding participant was subdivided into the ASD-NR group.

### Laboratory measurements

The venous blood was collected and the serum and cellular components were separated within 2 h after 3000 g centrifugation for 10 m. The obtained supernatant (serum) was stored at -80 °C for further analysis. The levels of total cholesterol (TC), triglyceride (TG), high-density lipoprotein cholesterol (HDL-C), and low-density lipoprotein cholesterol (LDL-C) were measured by the enzymatic method on Olympus AU5400 automatic biochemical analyzer (Olympus Ltd, Tokyo, Japan).

### Serum RBP4 measurements

Serum RBP4 levels were assayed in duplicate by using a sandwich enzyme-linked immunosorbent assay (ELISA) kit (R&D, Minneapolis, USA) according to the manufacturer’s protocol.

### Statistical analysis

IBM SPSS Statistics was used for data analysis. The normality of data distribution was determined with the Shapiro–Wilk test. Normally distributed data were expressed as (mean ± standard deviations). The skewness distributed variables were reported as the median (interquartile range). An independent sample t-test was used to compare the normally distributed continuous data between the two groups. Χ ^2^ test was used to compare the gender between the two groups. Mann-Whitney U test was used for statistical comparison of continuous data without normal distribution. Spearman’s rho test was used to analyze the correlations with the data of nonnormal distribution. Normally distributed data were tested using Pearson’s correlation. Logistic regression was used to analyze the risk factors for the autistic regression phenomenon, and the odds ratio (OR) and 95 % confidence interval (CI) were calculated. Crude OR and adjusted OR were estimated by bivariate and multivariate logistic regression analysis, and the crude model analyzed the crude data only, the adjusted model was adjusted for age, gender, BMI, TC, TG, LDL-C, HDL-C, CARS. Receiver operating characteristic (ROC) curve analysis was used to determine the optimum cut-off levels of RBP4 and TG to predict the autistic regression phenomenon. All statistical tests were two-tailed, and *p* < 0.05 was considered statistically significant.

## Results

### Characteristics of the participants

**Fig. 1:**
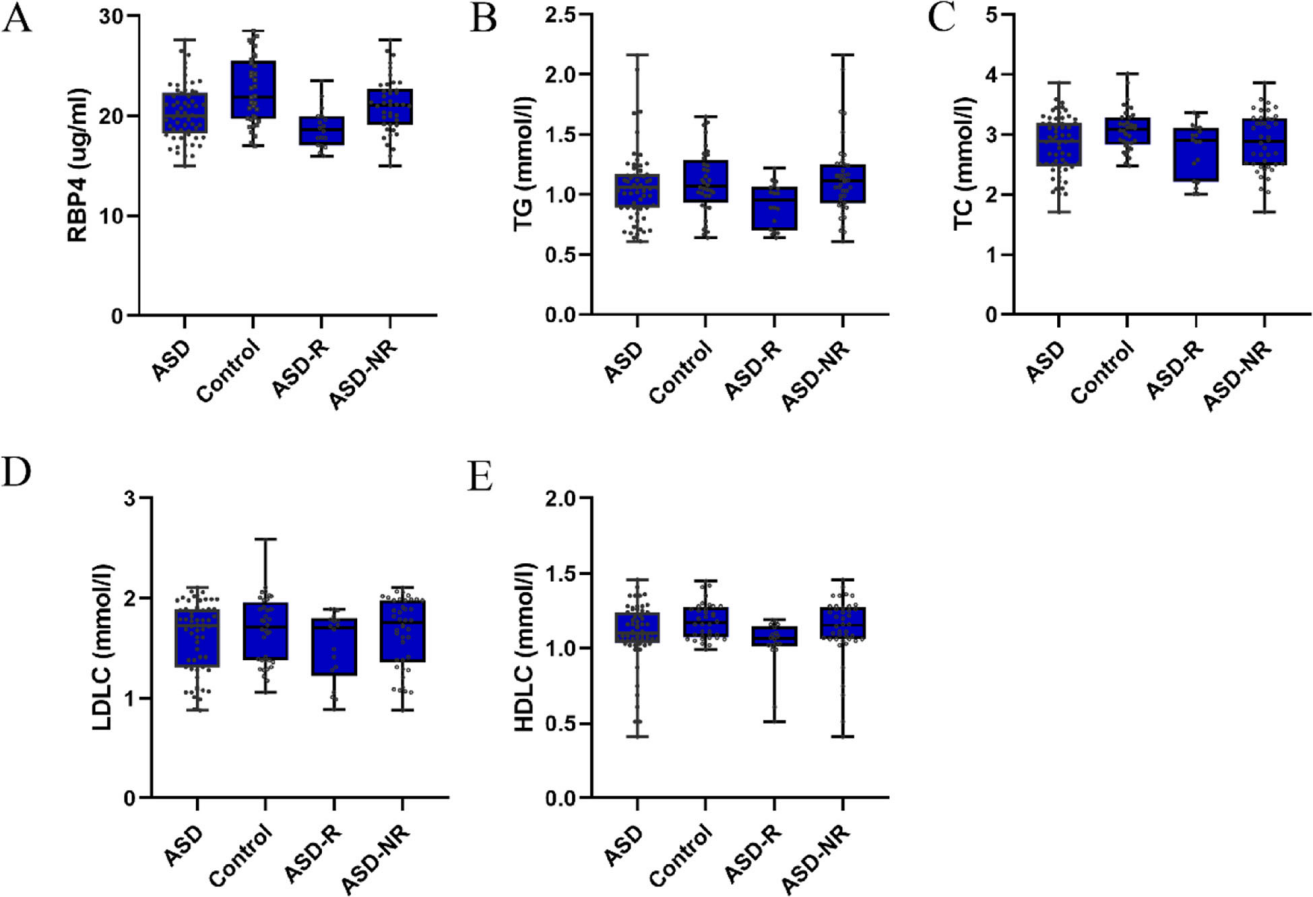
Distribution of all the laboratory measurements. Each box visually represents the laboratory data for the participants. RBP4: retinol-binding protein 4; TG: triglyceride; TC: cholesterol; LDLC: low–density lipoprotein; HDLC: high-density lipoprotein. ASD represents the autism group; Control represents the control group; ASD-R represents the autism group with the regression phenomenon; ASD-NR represents the autism group without the regression phenomenon

The demographic data and biochemical characteristics are presented in Table 1. The distribution of all the laboratory measurements is shown in Fig. 1. Compared with control subjects, autistic participants had lower levels of TC and RBP4 (*P* < 0.01), and a lower level of HDLC (*P* = 0.027).

**Table 1:** Characteristics of the participants

Variables	ASD	Control	*P* value	ASD(*n* = 60)		*P* value
	(*n* = 60)	(*n* = 36)		with regression	without regression	
Age(years)	7.27 ± 3.21	7.28 ± 3.12	0.987	7.56 ± 3.36	7.14 ± 3.17	0.65
Gender(male/female)	39/21	24/12	0.778	11/7	29/13	0.55
BMI	15.58 ± 1.17	15.32 ± 1.16	0.304	15.35 ± 0.07	16.50 ± 0.42	0.063
CARS	38.83 ± 8.25	16.00 ± 3.83	0	38.33 ± 9.13	39.05 ± 7.94	0.761
TG (mmol/l)	1.07 ± 0.30	1.10 ± 0.27	0.584	0.92 ± 0.18	1.13 ± 0.32	0.01
TC (mmol/l)	2.83 ± 0.48	3.08 ± 0.35	0.007	2.75 ± 0.46	2.87 ± 0.49	0.375
HDL-C(mmol/l)	1.10 ± 0.21	1.18 ± 0.12	0.027	1.02 ± 0.18	1.13 ± 0.22	0.088
LDL-C(mmol/l)	1.62 ± 0.35	1.68 ± 0.34	0.374	1.52 ± 0.35	1.66 ± 0.35	0.175
TG/TC	0.38 ± 0.12	0.36 ± 0.77	0.221	0.34 ± 0.79	0.40 ± 0.12	0.052
TG/HDLC	1.02 ± 0.40	0.93 ± 0.22	0.222	0.93 ± 0.27	1.06 ± 0.43	0.237
TG/LDLC	0.69 ± 0.25	0.67 ± 0.16	0.602	0.64 ± 0.23	0.71 ± 0.25	0.317
TC/HDLC	2.69 ± 0.74	2.62 ± 0.25	0.575	2.78 ± 0.77	2.65 ± 0.73	0.512
TC/LDLC	1.79 ± 0.28	1.87 ± 0.23	0.169	1.85 ± 2.69	1.77 ± 2.91	0.348
RBP4(ug/ml)	20.31 ± 2.79	22.56 ± 3.40	0.001	18.81 ± 1.99	21.08 ± 2.81	0.001

Variables are presented as mean ± standard deviation. *P*-values were derived from the independent sample t-test, Mann-Whitney U test, or χ2 test

*ASD* autism spectrum disorders, *RBP4 *retinol-binding protein 4, *TC* cholesterol, *HDLC* high-density lipoprotein, *LDLC* low–density lipoprotein, *TG* triglyceride, *CARS* Childhood Autism Rating Scale, *ADI-R *Autism Diagnostic Interview-Revised, *ASD-R* autism spectrum disorders with regression, *ASD-NR* autism spectrum disorders without regression, *ELISA* enzyme-linked immunosorbent assay, *BMI* Body Mass Index

Sixty autistic participants were subdivided into 18 participants with regression phenomenon and 42 individuals without regression phenomenon. Compared with those without regression phenomenon, individuals with regression phenomenon had lower TG and RBP4 (*P* < 0.01).

### Correlation of serum RBP4 with clinical parameters in autistic participants

The correlation of RBP4 with clinical parameters in autistic participants are shown in Table 2. In ASD-R group, the levels of RBP4 were positively correlated with TG (*r* = 0.474, *P* = 0.047) and TG/LDHC (*r* = 0.482, *P* = 0.043), were negatively correlated with CARS (*r *= -0.902, *P* < 0.001). In ASD-NR group, the levels of RBP4 were also positively correlated with TG (*r* = 0.364, *P* = 0.018) and TG/LDHC (*r* = 0.405, *P* = 0.008), were negatively correlated with CARS (*r* = -0.29, *P* = 0.004). In the whole ASD group, the levels of RBP4 were positively correlated with TG (*r* = 0.355, *P* = 0.005), HDLC (*r* = 0.257, *P* = 0.047), TG/TC (*r* = 0.376, *P* = 0.003), TG/LDLC (*r* = 0.363, *P* = 0.004), were negatively correlated with CARS (*r* = -0.296, *P* = 0.003).

**Table 2:** Correlation of serum RBP4 with clinical parameters in ASD individuals

	ASD with regression (*n* = 18)		ASD without regression (*n* = 42)		ASD (*n* = 60)	
	r	*P*	r	*P*	r	*P*
CARS	-0.902	0	-0.29	0.004	-0.296	0.003
Age(years)	0.273	0.273	0.156	0.324	0.134	0.306
Gender(male/female)	0.308	0.214	-0.23	0.142	-0.101	0.445
BMI	-0.165	0.513	-0.102	0.521	-0.129	0.326
TG	0.474	0.047	0.364	0.018	0.355	0.005
TC	0.163	0.518	0.028	0.861	0.079	0.547
HDL-C	0.223	0.375	0.156	0.324	0.257	0.047
LDL-C	-0.224	0.372	-0.122	0.44	-0.049	0.707
TG/TC	0.447	0.063	0.283	0.069	0.376	0.003
TG/HDLC	0.323	0.192	0.105	0.509	0.187	0.153
TG/LDLC	0.482	0.043	0.405	0.008	0.363	0.004
TC/HDLC	0.029	0.909	-0.239	0.128	-0.228	0.08
TC/LDLC	0.441	0.067	0.149	0.364	0.204	0.119

### Correlation of serum RBP4 and TG with autistic participants

**Fig. 2:**
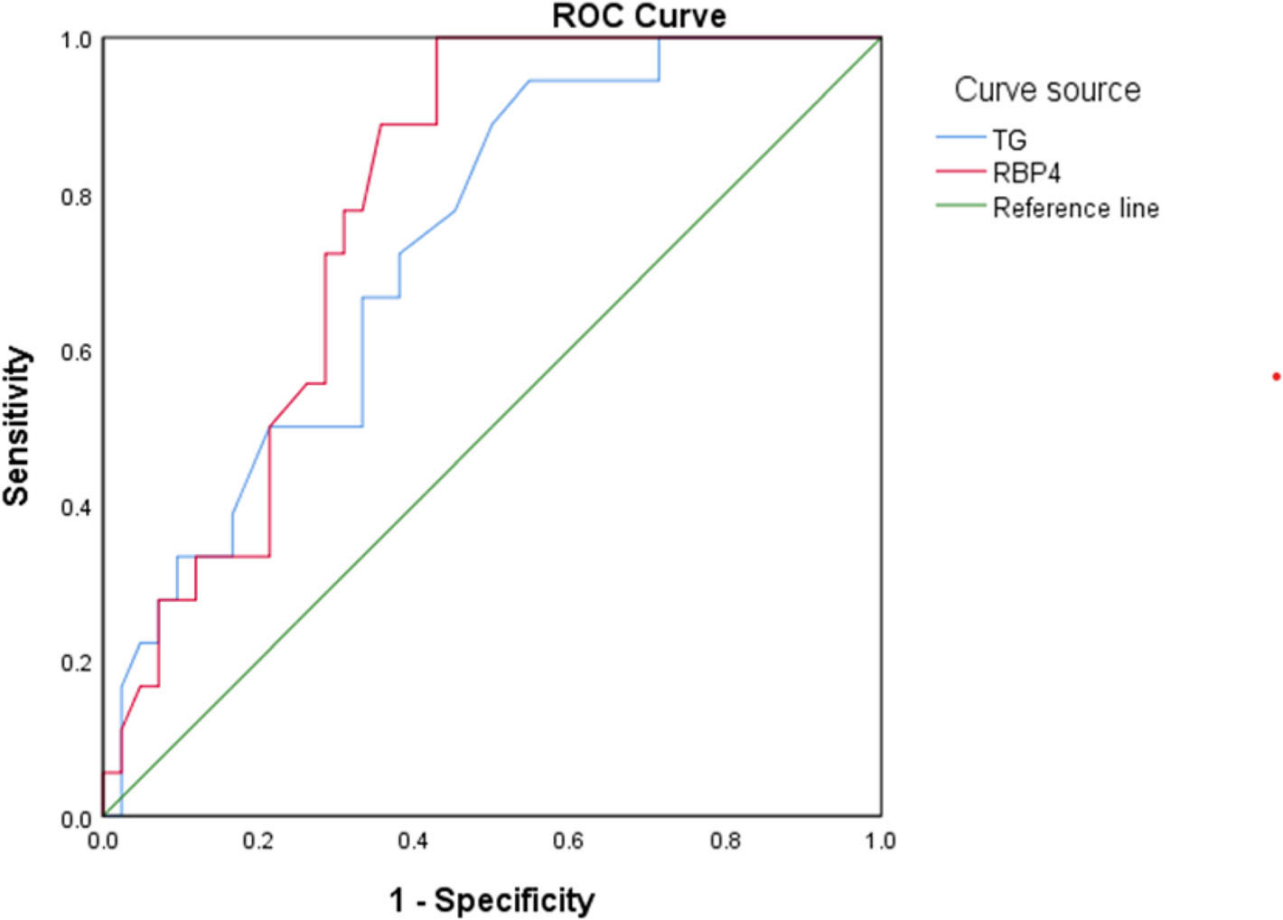
Receiver operating characteristic curves for the prediction of the autistic regression phenomenon by TG and RBP4. TG: triglyceride; RBP4: retinol-binding protein 4. The Area Under the Curve (AUC) is 0.782 for RBP4 and 0.733 for TG

As shown in Fig. 2, ROC curve analysis showed that the optimal cut-off value of TG for the prediction of the autistic regression phenomenon was 1.13 mmol/L, with a sensitivity of 94.4 % and a specificity of 39.7 % (area under the curve = 0.733, 95 %CI = 0.60–0.86, *P* = 0.005). In addition, the optimal cut-off value of RBP4 for the prediction of the autistic regression phenomenon was 20.6 ug/ml, with a sensitivity of 94.4 % and a specificity of 42.9 % (area under the curve = 0.782, 95 %CI = 0.67–0.89, *P* = 0.001).

After the adjustment of the potential confounding factors including age, gender, BMI, CARS, and the levels of TG, TC, HDL-C, LDL-C, logistic regression demonstrated that decreased RBP4 concentration increased the risk of the autistic regression phenomenon (OR = 1.59, 95 % CI: 1.10–2.29) (Table 3).

**Table 3:** Associations of serum RBP4 with ASD

		OR	95 %CI	*P* value
Crude model		1.573	1.169–2.115	0.03
Adjusted model		1.65	1.076–2.531	0.022

The adjusted model was adjusted for age, gender, BMI, TC, TG, LDL-C, HDL-C, CARS

*OR* odds ratio, *CI* confidence interval

## Discussion

The present study demonstrated that autistic participants had lower levels of TC, RBP4, and HDLC. Additionally, autistic participants with regression phenomenon had lower levels of TG and RBP4. Furthermore, the levels of RBP4 were positively correlated with TG and were negatively correlated with the severity of autism. Moreover, decreased RBP4 concentration increased the risk of the autistic regression phenomenon.

Consistent with the present study, some researchers also found that there were significant differences in TG, TC, HDL-C, non-HDL-C, and TC/HDL ratio in ASD group and healthy volunteers regardless of their diets [25, 26]. Differently, they showed increased levels of TC and BMI for ASD individuals, one possible reason for this difference could be the different age range. Interestingly, a nested case-control study demonstrated that lower maternal postpartum plasma LDL concentration was associated with increased odds of ASD in offspring [27]. Moreover, a retrospective case-control study of a group of French-Canadian ASD individuals observed four times more hypocholesterolemia in ASD than in the general population [28]. Furthermore, low TC in ASD was associated with higher rates of ASD-associated intellectual disability and anxiety/depression [28]. These results suggest that decreased level of TC may be related to ASD. However, it is difficult to judge whether cholesterol reduction is a cause or a consequence of ASD.

When compared with those without regression phenomenon, the present study demonstrated that autistic participants with regression phenomenon had lower TG. However, the present study didn’t detect the significant difference of TC or HDLC between ASD-R and ASD-NR groups. This indicates that TG may be related to the autistic regression phenomenon, whereas TC and TG may play different roles in the progression of the autistic regression phenomenon. Similarly, some researchers have found that mainly amino acid, lipid, and nicotinamide metabolism were significantly different between ASD with regression phenomenon group and ASD without regression phenomenon group [29]. In general, these results indicate that lipids may be related to the autistic regression phenomenon. Nowadays, researchers have demonstrated that fatty acid correction may serve as a treatment strategy for autism, especially for their core symptoms [19, 30, 31]. Therefore, it is speculated that correcting lipids may also improve regression in autism.

Former studies have found that Serum RBP4 levels of major depressive depression individuals were significantly lower than that of the healthy control group [32]. However, to our knowledge, the relationship between serum RBP4 level and ASD hasn’t been expounded. The present study first demonstrated that autistic participants had lower levels of RBP4 in comparison with the healthy controls, and the ASD-R group had lower levels of RBP4 compared with the ASD-NR group. Besides, the present results suggest that serum RBP4 concentration showed a negative correlation with the severity of ASD symptoms, subjects with decreased serum RBP4 levels were correlated with a nearly 1.65-fold increase in the risk of autistic regression phenomenon. This indicates that RBP4 may be related to the autistic regression phenomenon. As RBP4 is permeable to the BBB which is usually dysfunctional in most autistic participants [16], decreased serum RBP4 could be accompanied by a much higher brain level in ASD.

### Study strength

The results provide evidence that lipids and RBP4 were related to the presence and regression phenomenon of ASD. They can jointly predict the early diagnosis of ASD, guide early clinical intervention, early identification of regression phenomenon, and evaluate the prognosis of ASD.

### Limitations

The present study observed a significant difference in the serum level of RBP4 in ASD and healthy controls for the first time as well as the correlation between RBP4 and lipids. Nevertheless, several limitations of the present study should also be considered. As the study was performed in the Chinese Han population, the results need to be confirmed in other regions and ethnicities. In addition, the relatively small sample size may cause a potential low power of our results. Besides, the age range of the subjects included in this study was 3 to 14, whether those autistic participants who are out of this age range have similar characteristics is not clear. Further study is needed to expand the age range to demonstrate the changes in lipid and RBP4 throughout the life cycle of autistic participants.

## Conclusions

In summary, autistic participants had lower levels of RBP4, TC, and HDLC. Furthermore, compared with those autistic participants without regression phenomenon, autistic participants with regression phenomenon had lower TG and RBP4. Meanwhile, the levels of RBP4 were positively correlated with TG and were negatively correlated with CARS. Moreover, decreased RBP4 concentration increased the risk of the autistic regression phenomenon. Altogether, these results suggest that the serum RBP4 in combination with lipids may be considered predictors associated with the autistic regression phenomenon. Further studies are needed to expound whether decreased RBP4 participates in the development of the autistic regression phenomenon. The results indicate that correcting lipids and RBP4 to appropriate levels may help clinicians and practitioners in early intervention of the autistic regression phenomenon. However, it is important to note that the results must be interpreted with caution. Due to the heterogeneity of the condition, as well as the huge difference in developmental trajectories, the intervention should be tailored to that particular child regardless of being in a possible regression-risk group or not.

## Data Availability

Data and materials would be supplied based on reasonable requests.

## Abbreviations

ASD: Autism spectrum disorders
RBP4: Retinol-binding protein 4
BBB: Blood-brain barrier
GDM: Gestational diabetes mellitus
TC: Cholesterol
HDLC: High-density lipoprotein
LDLC: Low--density lipoprotein
TG: Triglyceride
CARS: Childhood Autism Rating Scale
ADI-R: Autism Diagnostic Interview-Revised
ASD-R: Autism spectrum disorders with regression phenomenon
ASD-NR: Autism spectrum disorders without regression phenomenon
ELISA: Enzyme-linked immunosorbent assay
BMI: Body Mass Index
